# History of the Royal Medical Society for the Years 1777 and 1778; with the Memoirs Relative to Physic and Medical Philosophy for Those Years, Taken from the Registers of the Society

**Published:** 1783

**Authors:** 


IV. *Hiſtoire de la Société Royale de Medecine, Années* 1777 *et* 1778. *Avec les Memoires de Medecine et de Phyſique Medicale, pour les memes années, tirés des Regiſtres de cette Société.* i. e. *Hiſtory of the Royal Medical Society for the Years* 1777 *and* 1778; *with the Memoirs relative to Phyſic and Medical Philoſophy for thoſe years, taken from the Regiſters of the Society.* 4to. Paris, 1780. 648 pages, with 3 copper-plates.

THE ſame mode of arrangement is obſerved in this as in the former volume. The eulogies of deceaſed aſſociates are thoſe of Linnæus, M. Arnaud de Nobleville, Dr. Macbride, and M. Barbeu Dubourg.

Of Louis Daniel Arnold de Nobleville, late preſident of the College of Phyſicians at Orleans, we are told that he was born in that city December 24th 1701. In conjunction with M. de Salerne, he reviſed and publiſhed M. Geoffroy's Materia Medica. He was likewiſe author of a book of Formulæ, and editor of M. Ferrein's Lectures on the Practice of Phyſic. But the work which gained him the moſt reputation was a treatiſe on the Nightingale, in which are a variety of curious obſervations relative to the natural natural hiſtory of that bird, the methods of taking it, feeding it, making it ſing, curing its diſeaſes, &c. M. de Nobleville was in affluent circumſtances, and his life is ſaid to have been a ſeries of benevolent actions. He died of apoplexy January 29th 1778.

The eulogy of the late Dr. David Macbride exhibits a very judicious review of his writings. This celebrated phyſician was born April 26, 1726, at Ballymony in the county of Antrim. His father, the Rev. Robert Macbride, was miniſter of a Preſbyterian congregation at that place. His mother was a daughter of Mr. Boyd of Killabeg, in the county of Down. After ſerving as a ſurgeon in the Royal Navy, he fixed at Dublin as a ſurgeon and accoucheur in 1749, but he did not acquire any conſiderable reputation till the year 1764, when he publiſhed his Experimental Eſſays, and was honoured with the degree of M. D. by the Univerſity of Glaſgow. From that period till his death, which happened after a ſhort illneſs on the 28th of December 1778, he was in extenſive practice. To great knowledge of his profeſſion and ſingular urbanity of manners, he united a taſte for the fine arts, particularly for Painting and Muſic. He was twice married, and had ſeveral children, all of whom whom died young. His brother, John Macbride, the only male of the family now living, is a captain in the Royal Navy, of diſtinguiſhed reputation for his bravery.

As a writer of eulogies, M. Vicq D'Azyr ſeems to rival M. de Fontenelle. There is ſo much elegance in the concluding paſſage of the preſent one in honour of Dr. Macbride, that we cannot refrain from giving it to our readers, tho' it loſes much of its ſpirit in a tranſlation.—

" The mothers of families—ſays the eulogiſt—

" watered his tomb with their tears; the poets

" ſtrewed it with flowers; his fellow-citizens

" honoured him with their praiſes: to complete

" his glory, there was wanting only that he

" ſhould be commemorated by his brethren

" amidſt the din of war, and beyond the ſeas,

" which, tho' they divide empires, afford no

" other barrier to the intercourſe of philoſophers

" than what ariſes from diſtance, and this is

" eaſily overcome by their genius and labours."

From the eulogy of James Barbeu Dubourg, we learn, that this phyſician was born at Mayenne February 15, 1709. He at firſt intended to go into the Church, and with this view acquired a critical knowledge of Hebrew; but he never took orders. It was not till the year 1748 that he he was admitted into the College of Phyficians at Paris. He was the friend of Lord Bolingbroke, and afterwards of Dr. Franklin. He tranflated Bolingbroke's letters on hiftory, and the whole of Dr. Franklin's works into French. For three years, viz. from 1761 to 1763, he was the editor of a weekly medical paper called *Gazette d'Epidaure*. He was likewife the author of *Recherches fur la durée de la groffeffe*, 8vo. *Le Botanifte François*, 2 vols. 12mo. and *Aphorifmes de Medecine*. This laft work has been publifhed fince his death, which happened on the 13th of December 1779.

*Meteorological Obfervations*.——Under this head Father Cotte (curate of Montmorency near Paris) has with wonderful induftry reduced into tables a view of the ftate of the thermometer, barometer, fall of rain, winds, difeafes, &c. in 96 different places in France, Holland, Switzerland, Germany, and Italy, during the years 1777 and 1778.

*Medical Topography*.——This article relates to the mountains of La Voge, a chain of mountains that extends 50 leagues from N. to S. and feparates Lorraine from Franche Comté. Thefe mountains give rife to feveral mineral fprings, which are here defcribed, and amongft

 others others to thofe of Plombieres, at the fouthern extremity of Lorraine, 17 leagues from Nancy. Thefe hot waters were not in vogue till the year 1614, when Henry II. Duke of Lorraine, found relief from them in fome ftomach complaints. This paper is written by M. Didelot, furgeon at Remiremont in Lorraine.

*Epidemics.*——Under this head we have, 1. *An account of an epidemic that prevailed in* 1774 *amongft the troops at Perpignan. By* M. Bonafos, phyfician at Perpignan.—2. *Of a fatal epidemic that prevailed in Ile-Jourdain, near Auch, in* 1777. *By* M. la Peyre, phyfician at Auch.—Ile-Jourdain is a marfhy fituation, and the fever here defcribed was of the remittent kind.

*Difeafes of Animals.*——This article contains, 1. *An account of a diforder that prevailed amongft the deer in* 1776 *in the foreft of St. Germains.*—2. *Remarks on the difeafes of the cattle in Poitou.*—3. *On the difeafes of fheep.* Thefe three are by Committees of the Society.—4. *An inquiry relative to the advantages which have been produced in Holland and Germany, by inoculating the difeafe of the horned cattle. By* M. Vicq D'Azyr. From thefe inquiries it appears that neither the dog, cat, horfe, or even ftag, although the latter is a ruminant ruminant animal, are capable of receiving this contagion by inoculation.

*Practice of Physic.*——1. *Observations on the inoculation of the small-pox.* These observations, which are communicated by different correspondents of the Society in France, who relate their success, may be useful in a country where this salutary practice still has prejudices to overcome. —2. *Case of a particular kind of colic and constipation. By* M. Lorry. We have here an account of a lady who was for a long time subject to pains in her bowels and obstinate costiveness. She gradually lost her strength and flesh, and at length died. On dissection, about a pint of water was found in the abdomen. The appearance of the viscera was natural, but the stomach was found divided into two distinct bags, communicating with each other.—3. *Inquiries relative to the miliary fever and its treatment, extracted from a second memoir by* M. Barailon. (See our 3d vol. p. 293.)—4. *Observations on a disease similar to the angina polyposa, or croup of children. By* M. Mahon, physician at Chartres. Two cases are related, both of which terminated fatally. No new lights are thrown on the disease. The author thinks that the membrane lining the trachea is the effect, not of inflammation of that tube, but of pus in the bronchi.—5. *Case of a suppuration of the liver, to the cure of which the use of cherries seemed to have contributed not a little. By* M. Desperrieres. A young man was attacked with all the symptoms of inflammation of the liver. A suppuration took place, and matter was voided in abundance both by stool and expectoration. On the 52d day he expressed a strong desire for cherries. He ate a pound a day, for upwards of a fortnight, and gradually recovered.—6. *Case of a dropsy of the liver. By* M. Caille. In this case the patient complained of a soft indolent tumour, of about the size of an hen's egg, on the right side of the umbilical region. It was at first supposed to be a lymphatic tumour of the integuments. Some time afterwards he experienced great pain in the region of the liver, and was attacked with a sort of tympany. His urine was in small quantity, and high coloured. These symptoms were followed by an ascites, and at the end of six weeks he was tapped. Twelve quarts of a thick, yellowish fluid were drawn off, and on the tenth day after the operation the patient died. On dissection, the abdominal cavity was found to contain a great quantity of the same fluid. The intestines adhered to each other, other, and on ſeparating them, about the region of the liver, a cyſt was diſcovered equal in bulk to the crown of a hat. It adhered to the concave ſurface of the great lobe of the liver, nearly where the gall-bladder is placed, but extended much farther under the great lobe towards the right ſide, ſo that it ſeemed to be formed by the gall-bladder, which was totally effaced, and by the membrane immediately inveſting the great lobe of the liver. This cyſt, which was externally of a white colour, excepting a few ſpots made by ſmall varicous veins, weighed about four pounds. It contained two quarts of a tranſparent ſerum. Its inner ſurface was lined with a thick membrane, evidently formed by coagulable lymph. At the bottom of the cyſt was a ſmall collection of hydatids. The great lobe of the liver was ſmaller than uſual. At its upper convex part was an abſceſs, containing a dark coloured bile. The ductus choledochus and hepatic ducts were effaced by the preſſure of the cyſt. The reſt of the viſcera were in a ſound ſtate.—7. *Caſe of a ſchirrus of the œſophagus, of the upper orifice of the ſtomach, and of the ſmall inteſtines. By* M. Carrere. A lady in her thirty-ſecond year was attacked with pain about the ſtomach, fever, coſtiveneſs, and vomiting. Theſe

ſymptoms

ſymptoms in the beginning were mitigated by mild evacuations and a ſuitable regimen, but at length returned with increaſed violence. The pains were acute, and the vomiting inceſſant. The patient loſt fleſh, and her ſtools became leſs and leſs frequent. She died in about two years. On diſſection, three or four quarts of a yellowiſh and very fœtid fluid were found in the abdomen. The pancreas was ſchirrous; the liver of its natural ſize, but of a pulpy conſiſtence; and the ſpleen very ſmall. The œſophagus, where it terminates in the ſtomach, and for above an inch upwards, had acquired the hardneſs of horn, and its cavity was nearly obliterated. The upper orifice of the ſtomach was ſchirrous, but the pylorus appeared to be in a natural ſtate. The ſmall inteſtines were ſchirrous, with their coats thickened, and their cavity contracted. The large inteſtines were in an inflamed ſtate.—8. *Caſe of a ſchirrous tumour of the œſophagus. By* M. Helian, phyſician at Metz. A man ſixty years old had for ſeveral years complained of difficulty of ſwallowing. The food paſſed the pharynx, but before it reached the ſtomach was vomited up. He died of atrophy. On diſſection, between the tunics of that part of the œſophagus which is behind the firſt diviſion of the the trachea, a ſchirrous tumour was diſcovered, three inches long and two in circumference, which almoſt totally cloſed the cavity of the gullet. The lungs and the ſtomach were diminiſhed to half their uſual bulk. The liver, pancreas, kidneys, and ſmall inteſtines were alſo leſſened, and the ſpleen weighed only three ounces.—9. *On the ſolvents of gall-ſtones.* *By* M. Durande. This phyſician, we are told, has communicated to the Society four more caſes, in which he has ſucceeded by a mixture of æther and ſpirit of turpentine. (See our 3d vol. p. 160.) M. de la Salle, it ſeems, by diſſolving theſe concretions in ſpirit of wine, and filtering the ſolution, has obtained a great quantity of a ſalt that reſembles ſedative ſalt.—10. *On the external uſe of different tinctures of cantharides.* *By* M. Andry. The friends of a patient being averſe to bliſters, tincture of cantharides was directed to be rubbed on his feet and legs. Inſtead of the common tincture, that of Fuller was by miſtake applied, and its effect was ſo quick and powerful, that for this uſe M. Andry prefers it to every other. —11. *An account of the treatment of four perſons who had taken verdigreaſe.* *By* M. Jeanroi. Two of theſe patients were relieved by clyſters, and drinking freely of milk; the other two, who were were more affected, were cured by an emetic, oily emulfions and clyfters.—12. *An account of a malignant carbuncle* (charbon malin). *By* M. Paulet. This is the anthrax of the ancient Greek writers. It begins in the form of a pimple, and is attended with an acute burning pain. The fkin round it fwells, inflames, and becomes of a fhining red colour, while the center appears of a livid hue. In proportion as the inflammation fpreads, the pulfe quickens, and febrile fymptoms are produced. This difeafe, we are told, is not contagious. At Paris it is obferved to be confined chiefly to tallow-chandlers, and manufacturers of horfe-hair. The latter are faid to be the moft frequently attacked with it. It feldom happens that they are long in the trade without having it, and many die of it. Thefe people have obferved, that the hair which is moft to be feared is that which comes from Ruffia. This is commonly in a bad ftate when it arrives, part of it being generally of a grey colour, and of a difagreeable fmell. A girl, whom M. Paulet faw labouring under the difeafe in queftion, had opened a parcel of this fort of hair. The carbuncle fhe had was perfectly fimilar to thofe which have been obferved in Languedoc in perfons who handle and wafh wool, or who fhear fheep.

ſheep. M. Paulet leaves it to farther experience to determine whether the matter, the contact of which produces this eruption, is the reſult merely of ſome change in an animal ſubſtance undergoing a kind of putrefaction, or whether the hair that is capable of exciting the diſeaſe in queſtion, has not been taken from horſes that have died of a ſimilar complaint.

[ *To be continued.* ]

